# A Fast and Scalable Kymograph Alignment Algorithm for Nanochannel-Based Optical DNA Mappings

**DOI:** 10.1371/journal.pone.0121905

**Published:** 2015-04-13

**Authors:** Charleston Noble, Adam N. Nilsson, Camilla Freitag, Jason P. Beech, Jonas O. Tegenfeldt, Tobias Ambjörnsson

**Affiliations:** 1 Department of Astronomy and Theoretical Physics, Lund University, Lund, Sweden; 2 Division of Solid State Physics, Department of Physics, Lund University, Lund, Sweden; 3 Department of Physics, Gothenburg University, Gothenburg, Sweden; Imperial College London, UNITED KINGDOM

## Abstract

Optical mapping by direct visualization of individual DNA molecules, stretched in nanochannels with sequence-specific fluorescent labeling, represents a promising tool for disease diagnostics and genomics. An important challenge for this technique is thermal motion of the DNA as it undergoes imaging; this blurs fluorescent patterns along the DNA and results in information loss. Correcting for this effect (a process referred to as kymograph alignment) is a common preprocessing step in nanochannel-based optical mapping workflows, and we present here a highly efficient algorithm to accomplish this via pattern recognition. We compare our method with the one previous approach, and we find that our method is orders of magnitude faster while producing data of similar quality. We demonstrate proof of principle of our approach on experimental data consisting of melt mapped bacteriophage DNA.

## Introduction

Optical mapping is an emergent complementary approach to DNA sequencing which produces a lower resolution (typically kbp) sequence-dependent map of individual DNA molecules [1–10]. As a direct complement to DNA sequencing, optical mapping can produce a scaffold to facilitate easier sequence assembly, and as an indirect complement, optical mapping promises a variety of applications for which low resolution maps will suffice. For example, it can be used to quickly identify large-scale structural variations, including duplications, deletions, insertions, inversions, and translocations, which are increasingly being linked to heritable traits of phenotypic significance [3, 11, 12], and it allows for the rapid identification of bacterial species and strains which could represent an important step against the growing problem of antibiotic resistance [13–17].

To date, many techniques for optical mapping have been developed, and they typically rely on sequence-specific DNA modifications at short target sites, followed by imaging and analysis. These sequence-specific modifications can include staining and denaturation (“melt mapping”) [18], fluorocoding [19], competitive binding of intercalating dyes [20, 21], methylation [22], enzymatic nicking [23, 24], and enzymatic restriction [25–27].

These optical mapping techniques can roughly be divided into three groups: stretching over a surface [9, 28–30], stretching via confinement in nanochannels [4, 31–36] or stretching via elongational flow in microchannels [37]. While surface–stretching techniques offer a few advantages, such as allowing for 100–140% extension [26], mapping via nanochannel confinement allows for integration of the stretching in a lab on a chip context that in turn can be brought to application much more easily.

**Fig 1:**
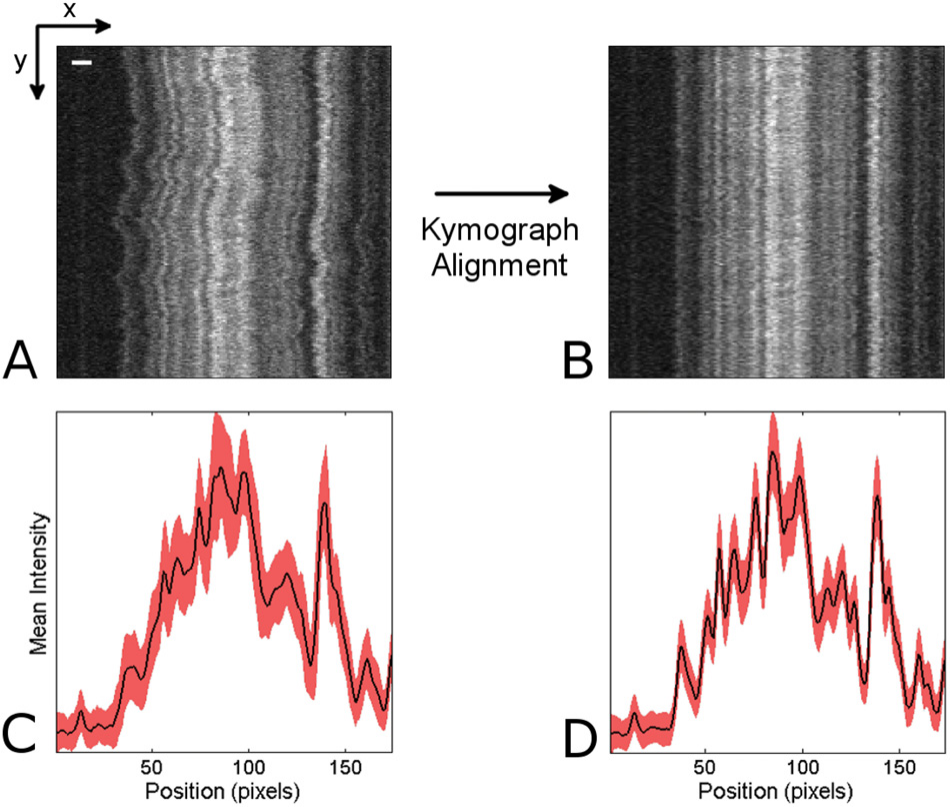
Problem definition. (A) A raw kymograph depicting denaturation mapping of an intact T4GT7 bacteriophage DNA molecule. The y-axis represents time (200 time frames over 20 seconds) while the x-axis represents the DNA extension in the nanochannel (170 pixels wide; scale bar is 2*μ*m). Note that these specifications also apply to all of the following kymographs. (B) The image from (A) after undergoing the kymograph alignment process. (C) Trace of mean intensity over time for the raw kymograph (black) with ±1 standard deviation (red), and (D) the same for the aligned kymograph.

Note that the nanochannel-based DNA barcoding schemes, the main focus of this study, should not be confused with schemes where short genetic markers [38] or restriction enzyme cleavage events [25] are detected as landmarks. Barcodes addressed by our technique are not simply binary in the sense that one detects landmarks or the absence thereof; rather what we call “barcodes” are continuous fluorescence profiles which are more susceptible to thermal noise (see Fig 1a).

One particular problem inherent to nanochannel confinement techniques is that DNA tends to undergo random diffusive processes during imaging, including center-of-mass diffusion and local stretching [39]. To correct for these effects, a procedure we denote *kymograph alignment* must be performed (see section Problem Definition). In this paper we present WPAlign (**W**eighted **P**ath **Align**), an algorithm for kymograph alignment which offers linear scaling in time and can align DNA barcodes with length corresponding to an entire human genome in less than an hour on a typical desktop computer. We compare its performance to an existing technique and show that our method offers orders-of-magnitude improvement in computational speed while producing processed data of similar quality. Additionally, we present a new information score which quantifies the information content of DNA barcodes and should see widespread use as a barcode quality criterion by which experimentalists can evaluate barcodes and optimize experimental mapping conditions.

## Problem Definition

In Fig 1a, the result of a typical nanochannel-based optical DNA mapping experiment is displayed. The horizontal axis (x-axis) of this kymograph represents the DNA’s extension in the nanochannel (where the pattern results from sequence-specific staining), and the vertical (y-axis) represents images of the DNA molecule at different times.

Due to thermal center of mass and local conformational fluctuations (which occur because the flexible DNA molecule is only extended to roughly 50 percent of its contour length), the images over time are misaligned both locally and at a global level, leading to significant noise during time-averaging (see Fig 1c). Thus before a useful time-average can be performed, the bright and dark bands must be “straightened”; this procedure we refer to as *kymograph alignment*. Fig 1b shows the result of our algorithm, WPAlign, developed for this particular task, and Fig 1d shows the corresponding time-average. The details of the algorithm can be found in the Methods section. Intuitively, the purpose of this process is to, as closely as possible, mimic the results which would be obtained if the DNA were held and stretched to 100 percent of its length during imaging. For practical experimental reasons, this is not feasible for nanochannel-based techniques, so one must correct for misalignments such as those displayed in Fig 1a.

In Ref. [18], the kymograph alignment challenge is mathematically presented as a global optimization problem (see S1 for details): a template time frame, *T*, is first chosen. One then allows for local stretching of all the remaining frames, *N*_*i*_, and maximizes the overlap between *N*_*i*_ and *T*. Finally, all the time frames are rescaled with average global stretching factors in order to ensure that the end-to-end distance of the DNA molecule after alignment is unchanged and that the time-averaged optical map is therefore consistent with thermodynamic constraints.

In this study we pose the kymograph alignment challenge in slightly different mathematical terms, namely as a feature detection problem (details found in Methods). Visually, our method (see Results) produces appealing images which are very similar to those produced using the previous method. The main benefit of our approach is that it provides an orders-of-magnitude reduction in computational time. Furthermore, all the substeps in our algorithm use standard numerical tools and are, therefore, straightforward to implement.

The rest of this study is organized as follows: in Methods we introduce our kymograph alignment algorithm, WPAlign. In Results, we compare our new algorithm to the previous method. In Discussion we summarize our results and point to further potential applications of our algorithm, and in the S1 we provide more technical details of our approach.

## Methods

We now consider the problem defined in the previous section, i.e., the alignment of a “fuzzy” DNA barcode (see Fig 1a for an illustrative example) distorted by thermal fluctuations as it resides in a nanochannel. WPAlign works intuitively by detecting pronounced bright or dark ridges (“features”) and then stretching the barcode horizontally to straighten them. The advantage of this approach is that it reduces the problem to a simple two-step process of (1) *single-feature detection*, followed by (2) *single feature alignment*. This two-step process is then be applied recursively to align the entire kymograph.

### Single Feature Detection

Consider an image represented as a 2D gray scale intensity function with columns *x* and rows *y*, denoted *I*(*x*, *y*), *x* ∈ [1, *n*], *y* ∈ [1, *m*]. See Fig 1a for an illustrative example. Then a feature *x* = *F*(*y*) is a function mapping of rows *Y* to columns *X*. This mapping thus provides a curve, (*x*, *y*) = (*F*(*y*), *y*), through the image. Intuitively the feature *F* we would like to detect is the “most pronounced” vertical ridge or valley in *I* such that:

1. (Completeness) Our detected feature *F* traverses each row *y* exactly once. Actual features might not adhere to this constraint, since anomalous events such as DNA breakage could occur, but these events should be treated separately from the alignment process.
2. (Continuity) *F*’s horizontal movement is constrained. I.e., |*F*(*y*+1) − *F*(*y*)| ≤ *k* for some integer *k* and for all *y*. This is to fit the physical constraints we inherit from the polymer physics of DNA molecules for the present application.

Unfortunately, standard feature detection (e.g., ridge detection) methods cannot be applied directly, as the resulting features will not necessarily satisfy these constraints, so we have developed the following three-step procedure for *single feature detection*:

#### 1. Image pre-processing

**Fig 2:**
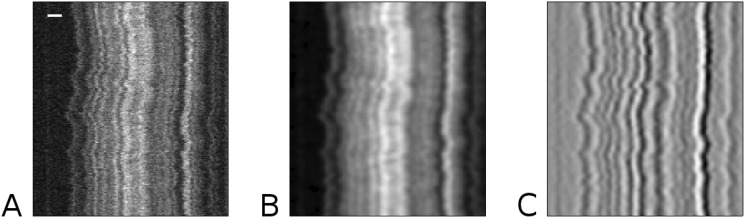
Image preprocessing. (A) The raw, unaligned, kymograph shown in Fig 1a (scale bar is 2 *μ*m, 20 seconds of imaging). (B) The kymograph after Gaussian smoothing, and (C) The Laplacian response image, which we refer to as *K*, that results from applying the Laplacian of Gaussian filter (with *σ* = 10 pixels) to our smoothed kymograph. In *K*, dark regions represent positive values and light regions represent negative values.

First the entire image is smoothed with a 2D Gaussian kernel (here we use *σ*_*h*_ = 10 pixels and *σ*_*v*_ = 3 pixels, where *h* and *v* represent the horizontal and vertical directions, respectively) to remove noise from random intensity fluctuations (see Fig 2b). Then we apply a Laplacian of Gaussian filter to obtain the *Laplacian response* image which we refer to as *K* (see Fig 2c), a matrix with the same size as *I* which has large positive values in dark bands and large negative values in light bands (see also S1). These positive and negative values are linearly rescaled to range from (0,1] and [−1,0), respectively.

**Fig 3:**
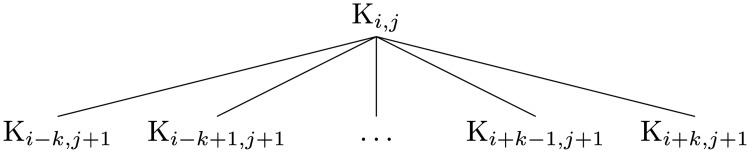
Connectivity between nodes.

**Fig 4:**
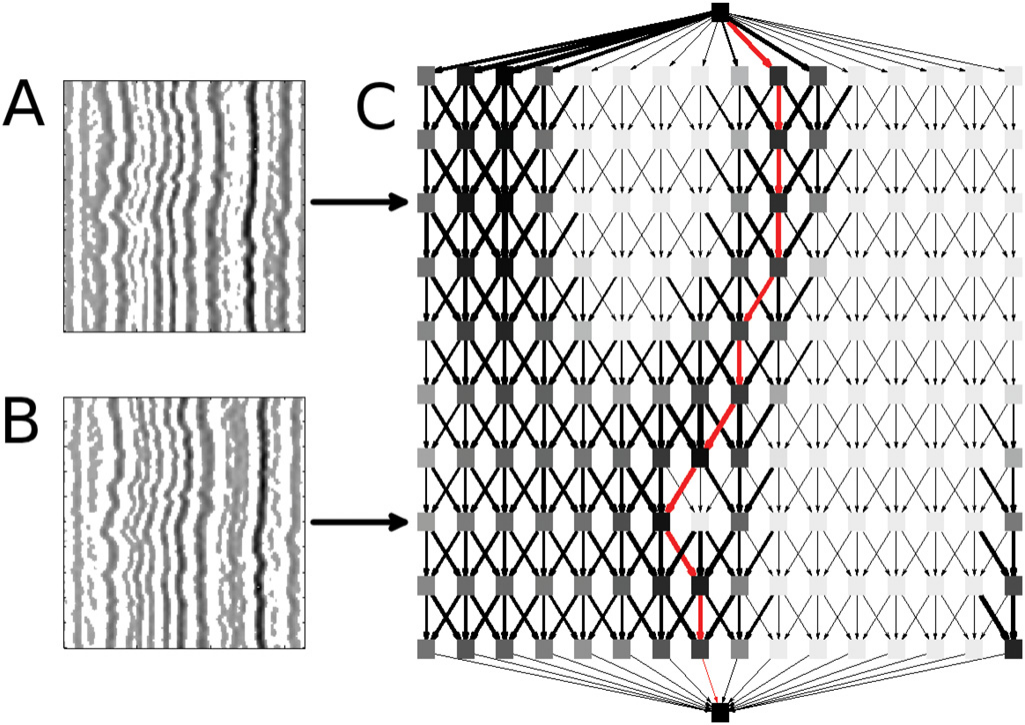
Network assembly. The Laplacian response image, *K*, (see Fig 2c) has been rescaled into (A) *K*_*D*_ and (B) *K*_*B*_, images that emphasize dark and bright regions in *K*, respectively. White pixels represent barriers that potential features cannot cross, while continuous dark regions indicate likely features. (C) Here we show one example network, although separate networks are indeed assembled for *K*_*B*_ and *K*_*D*_. Each node (square) within the rectangular region represents a pixel in K_*B*_ or *K*_*D*_. The top and bottom nodes (which we term “peripheral nodes”) are added to provide starting/ending points for the shortest path finding algorithm. The width of the edges corresponds to the inverse of the edge weight, and the darkness of the nodes represents the average weight of incoming edges. The red line illustrates the shortest path through the network. For the sake of visual clarity, this network was created using a small subsection of an actual Laplacian response with *k* = 1.

Now our feature *F* should be represented by a continuous region of high (valley) or low (ridge) values in *K*. We would like to treat these as two distinct cases to avoid detecting a single feature which is partially composed of a ridge and partially composed of a valley, so we compute two separate images, *K*_*B*_ and *K*_*D*_ which emphasize bright and dark regions, respectively (see Fig 3, Fig 4 and S1). Both are positive or zero everywhere, with the smallest values representing the most pronounced feature locations.

#### 2. Network assembly

We can think of the images *K*_*B*_ and *K*_*D*_ as energy landscapes, and finding the best ridge or valley becomes a problem of finding the lowest-energy paths through *K*_*B*_ and *K*_*D*_, respectively. To do this, we assemble directed, acyclic networks *G*_*B*_ and *G*_*D*_ as follows (see Fig 4). For simplicity, we describe our process only for *G*_*B*_, as the process is identical for *G*_*D*_.

1. First, we create one node for every pixel in *K*_*B*_, plus two “peripheral” nodes. Thus *G*_*B*_ consists of (*m* × *n*+2) nodes.
2. Now the first of the peripheral nodes is connected to each of the nodes corresponding to the first row of *G*_*B*_, and each of the last-row nodes is connected to the second peripheral node with edge weight 1.
3. The rest of the nodes are connected as follows: the node corresponding to pixel (*i*, *j*) in *K*_*B*_ is connected to pixels in the next row directly below, to the left *k* columns, and to the right *k* columns (see Fig 3). This *k* value is used to satisfy the continuity constraint above. We have found *k* = 2 to be reasonable for our current application.
4. If a pixel is too close to a border on the left or right (i.e., *i* − *k* < 1 or *i*+*k* > *n*), connections to non-existing nodes prescribed in step 3 are ignored.
5. Finally, every edge is assigned a weight equal to the intensity of the pixel in *K*_*B*_ corresponding to the node it is directed to.

Note that the edge weights in this connection scheme are not “biased,” in the sense that given equal intensities, movement to any of the connected pixels is equally likely. Alternatively, one could assign a non-uniform distribution such that the edge weights are given by a combination of intensities and some prior knowledge of likely fluctuations. For example, the weight of the edge connecting (*i*, *j*) to (*i*′, *j*′) could be given by *w*_*i* → *i*′,*j* → *j*′_ = *I*(*i*′, *j*′)*f*(*i*′, *i*), where *f* is some probability distribution. Our scheme here represents a special uniform case where *f*(*i*′, *i*) = 1/(2*k* + 1) for ∣*i*′ −*i*∣ ≤ *k* and *f*(*i*′, *i*) = 0 otherwise.

After examining several additional forms for *f*, we found that the simple uniform case presented above achieved the best and most consistent results for our examples. However, this weighting scheme should, in general, be carefully chosen by the user to suit the particular data being considered.

As for the choice of *k*, we found *k* = 2 to be reasonable for the data presented here; now we present a method for choosing *k* to be used on novel data. For unbiased diffusive motion, the diffusion length Δ*L* of the DNA molecule is connected to time as Δ*L* ^2^ ∝ *Dt* (where *t* is the inverse sampling frequency), and the center-of-mass diffusion constant *D* is inversely proportional to the number of polymer segments (i.e., the length of the polymer)[40]. Hence $\Delta L\propto 1/\sqrt{L}$, where *L* is the length of the DNA molecule. For experimental setups identical to ours, the choice of *k* will vary based on the DNA length to be aligned according to:

$$\begin{array}{c} k=k_{\text{ref}}\sqrt{\frac{L_{\text{ref}}}{L}} \end{array}$$

where *k*_ref_ = 2, as presented here, and *L*_ref_ = 24*μm*, the length of the molecules presented above.

#### 3. Shortest path finding

Now every path between the peripheral nodes in our networks represents a potential feature which satisfies the continuity and completeness constraints above, so our task is to find the “best” such feature. Essentially, we are searching over all paths between the peripheral nodes which do not move more than *k* pixels horizontally between any adjacent rows. Our cost function is the sum of pixel intensities over a path, and we seek to minimize this over the space of these acceptable paths. Thus, intuitively, the best bright feature corresponds to the shortest path in *K*_*B*_, and the best dark feature corresponds to the shortest path in *K*_*D*_. So, our task becomes a shortest path finding problem.

Since our networks are directed and acyclic, the shortest path can be computed by a standard dynamic programming algorithm based on topological sorting which grows linearly with the sum of the number of edges and nodes [41]. Note that, in our graph, this sum grows bilinearly with the number of time frames and the horizontal width of our input kymographs. And since the number of time frames does not change, in practice, the sum of nodes and edges in our graph grows linearly with the kymograph width. Thus the computational time of the shortest path finding algorithm also grows linearly with the kymograph width.

By this process we obtain two paths, one in *K*_*B*_ corresponding to the best bright feature in our original kymograph, and one in *K*_*D*_ corresponding to the best dark feature. The path with the shorter length of these two is chosen as the best overall feature (see Fig 4). If this feature is distinct enough (i.e., its length is lower than some threshold; see Methods on calculating *K*_*D*_ and *K*_*B*_) then we proceed with alignment. Otherwise the path is rejected, and recursion is terminated. This can happen, for instance, in large dark ‘gaps’ associated with low labeling density mapping approaches.

### Single Feature Alignment

**Fig 5:**
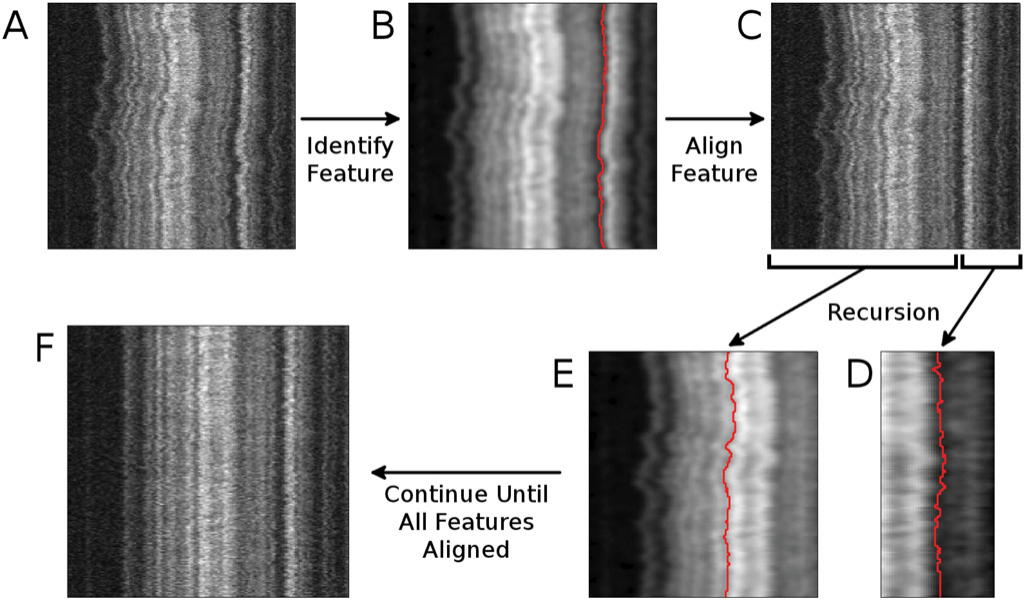
Feature detection and recursion in WPAlign. (A) A raw, unaligned kymograph is given as input (see Fig 1). (B) The “best” feature is identified from the input kymograph. (C) The feature identified in (B) is aligned via linear interpolation. (D, E) The feature identification process is called recursively on the regions to the right (D) and to the left (E) of the newly-aligned feature. (F) This process is continued until all features have been aligned.

Once the best feature *F*(*y*) has been detected by the three-step process above, it is aligned by setting *F*(*y*) = ⟨*F*(*y*)⟩, where ⟨.⟩ denotes the mean (rounding to the nearest integer) for all rows *y*, and the pixels to the left and right of *F*(*y*) are stretched (or compressed) linearly. To determine intensity values at non-integer positions during this linear stretching, we used cubic spline interpolation (see Fig 5). Note that by setting *F*(*y*) = ⟨*F*(*y*)⟩, we ensure that the feature is aligned over its position at thermodynamic equilibrium.

### Full Kymograph Alignment

Using the scheme above, the best feature in the input image has been both (1) *detected* and (2) *aligned*. But we wish to detect and straighten all of the features. Thus to continue the process, the image is split vertically along the newly aligned feature (see Fig 5c–5e), and *w* columns (where *w* is set to half the width of a typical feature) are removed from each on the side adjacent to the split. Then the (1) *single feature detection* and (2) *single feature alignment* routines are called again on each of these two smaller images if they are wider than typical a feature (i.e., 2*w*). In our current application, features are typically ∼ 10 pixels wide, so we use *w* = 5 pixels, though this parameter can easily be changed depending on the application. (see Fig 5). The algorithm then terminates when the width of the image is less than the width of a typical feature, 2*w*.

It is worth noting that, since we remove columns before calling the process again, the algorithm is guaranteed to converge. If we did not perform this step, the same feature could conceivably be re-straightened perpetually.

**Fig 6:**
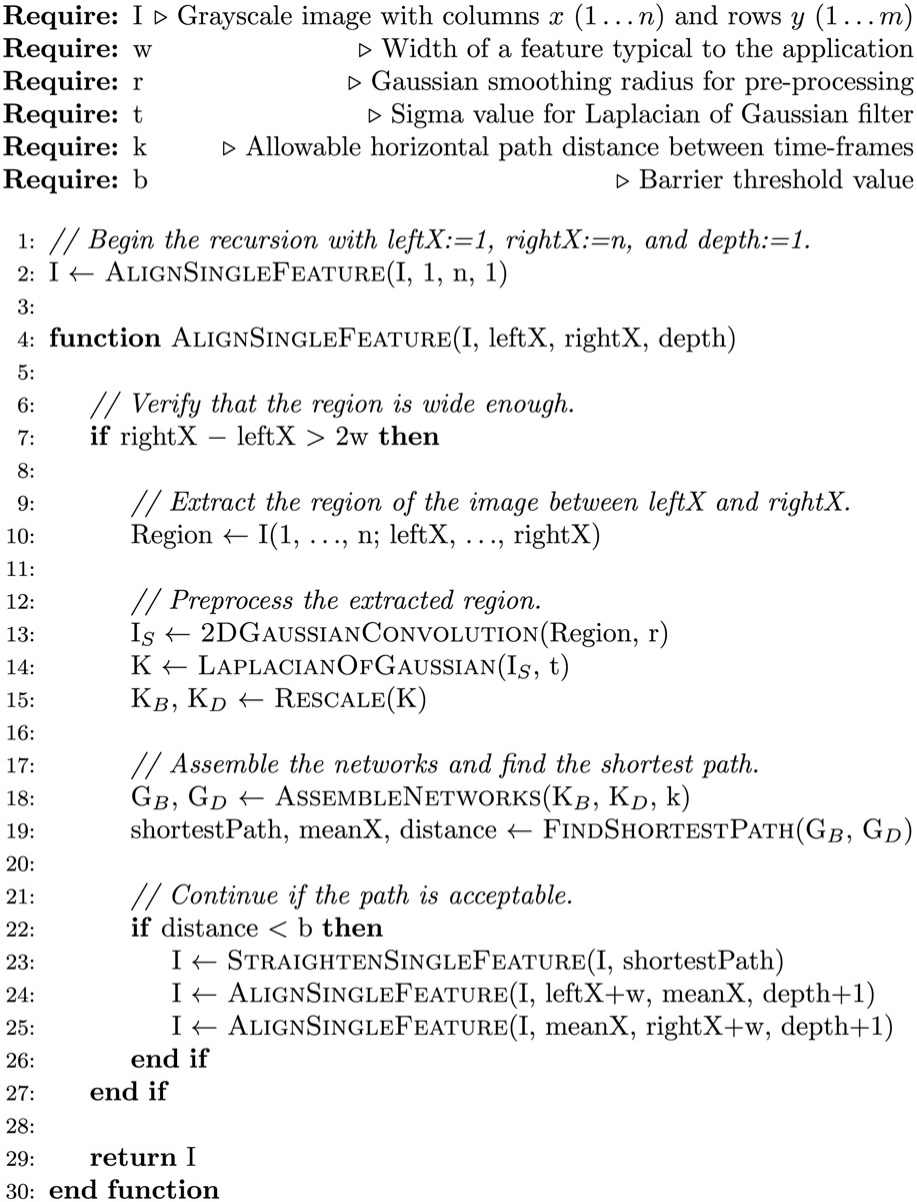
WPAlign pseudocode. Note that all substeps shown here are available in a variety of standard toolboxes, open-source and otherwise. For our particular implementation, all code was written in Matlab with dependencies in the Image Processing Toolbox and the Bioinformatics Toolbox (which implements the graph data structure and shortest path finding functionality).

A pseudocode describing all the steps in our kymograph alignment method, WPAlign, is found in Fig 6.

## Results

**Fig 7:**
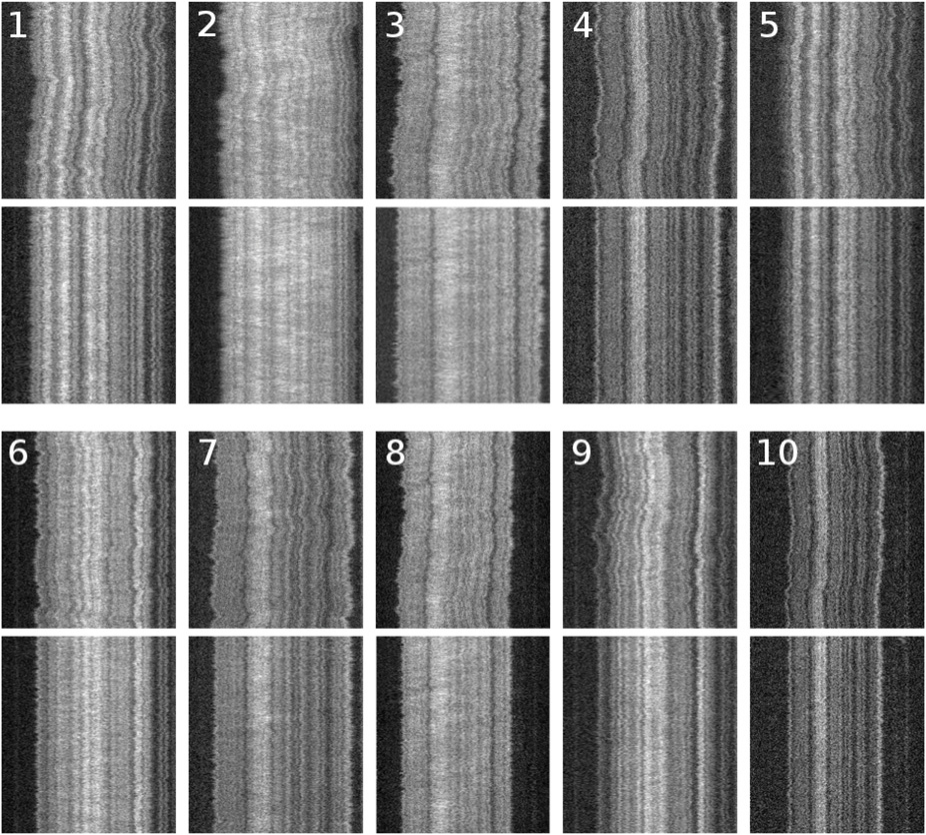
Typical T4GT7 denaturation mapping kymographs, raw and aligned via WPAlign. The numbered kymographs represent the raw data, and the aligned versions are displayed directly below each.

In practice, WPAlign produces alignments which are visually appealing (see Fig 7). To quantify the quality of these alignments, we use two quality criteria: *time-trace noise reduction* and *information content* along with empirical computational costs. We compare WPAlign to the method used in [18] (see S1 for a description) with respect to these measures.

### Time Trace Noise Reduction

The ultimate goal of presenting the data in a kymograph format is to produce a 1-dimensional intensity profile that is “typical” and reproducible for the DNA being analyzed. This intensity profile is then compared to existing databases for genomic analysis. To produce this intensity profile, ⟨*I*⟩, we simply take the time-average of an aligned kymograph

$$\begin{array}{c} {\left\langle I\left(x,y\right)\right\rangle }_{y}=\frac{1}{m}\sum_{y=1}^{m}I\left(x,y\right), \end{array}$$ (1)

where *m* is the number of rows (time frames) in the kymograph. The reduction in noise can be quantified by the column-wise variance, given by

$$\begin{array}{c} \sigma^{2}\left(x\right)=\frac{1}{m}\sum_{y=1}^{m}{\left[I\left(x,y\right)-{\left\langle I\left(x,y\right)\right\rangle }_{y}\right]}^{2}\cdot \end{array}$$ (2)

**Fig 8:**
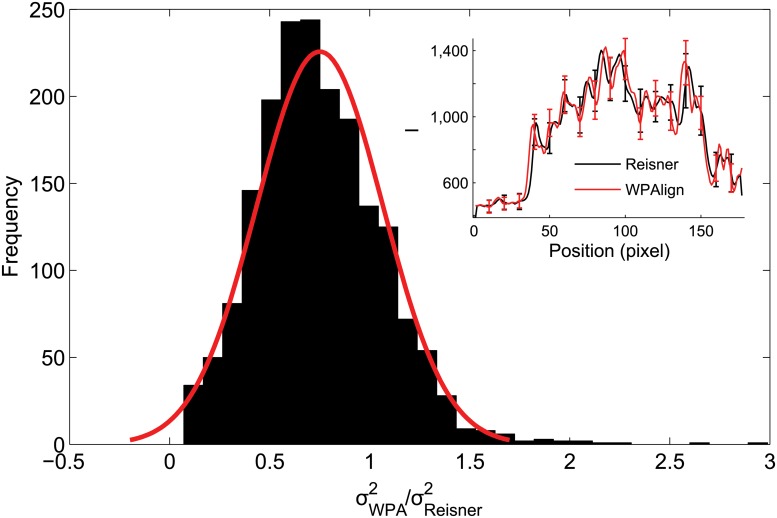
Kymograph noise comparison of WPAlign and the Reisner approach. Column-wise variances were calculated for aligned barcodes using WPAlign ($\sigma_{W}^{2}(x)$), and the Reisner approach ($\sigma_{R}^{2}(x)$). Here we show the distribution of $\sigma_{W}^{2}/\sigma_{R}^{2}$. A Gaussian distribution was fit to this data, resulting in a mean of *μ* = 0.75 (with standard deviation *σ* = 0.32). Thus in this particular case, WPAlign reduces variance by 25% compared to the Reisner method. Overall, the fraction of columns such that $\sigma_{W}^{2}<\sigma_{R}^{2}$ was 80%. (Inset) An example time trace of barcode 6 (see Fig 7) aligned via the WPAlign and Reisner methods. Error bars represent one standard deviation (i.e., *σ*_*W*_ and *σ*_*R*_).

To compare WPAlign and the Reisner method [18], these variances were calculated for the 10 representative kymographs in Fig 7, see S1 for experimental details on how these kymographs were obtained. For every column in each of the kymographs, we obtained values $\sigma_{W}^{2}(x)$ and $\sigma_{R}^{2}(x)$, corresponding to the variances resulting from alignment by the respective algorithms. Then a distribution of values $\sigma_{W}^{2}(x)/\sigma_{R}^{2}(x)$ was calculated (see Fig 8). This distribution is normal with mean *μ* = 0.75, indicating that, for a typical kymograph column, WPAlign reduced variance by 25% when compared to the Reisner method. Thus WPAlign, besides reducing computational costs, reduces kymograph noise and yields an improvement over the Reisner approach.

**Fig 9:**
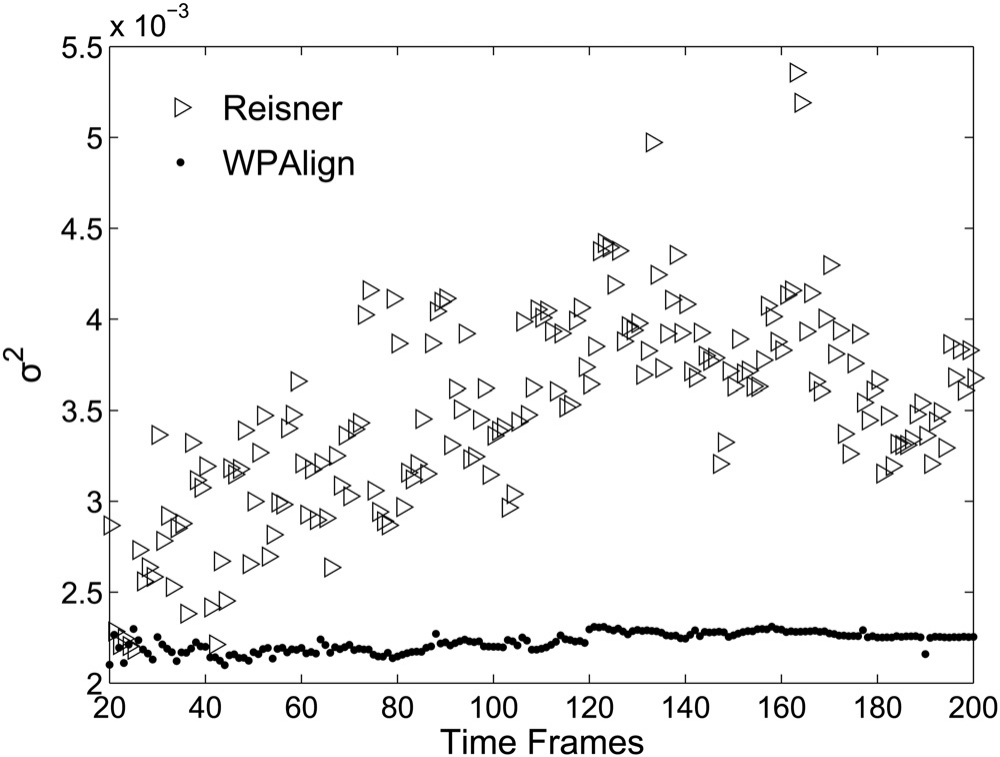
Effect of time axis length on aligned kymograph noise. Kymographs with time axes varying from 20 to 200 time frames in length were aligned by both methods. The resulting column-wise variances $\sigma_{R}^{2}(x)$ and $\sigma_{W}^{2}(x)$ were calculated as in Fig 8. Here we plot, for each kymograph, the mean of these column variances, i.e., $\left\langle \sigma_{R}^{2}(x)\right\rangle$ and $\left\langle \sigma_{W}^{2}(x)\right\rangle$, showing that kymograph noise increases with time axis length for the Reisner method but remains constant for WPAlign.

In addition, we examined how aligned kymograph noise is affected by the length of the time axis (see Fig 9). To do this, we calculated ⟨*σ* ^2^(*x*)⟩, the mean of the column-wise variances given above, given by

$$\begin{array}{c} \left\langle \sigma^{2}\left(x\right)\right\rangle =\frac{1}{n}\sum_{x=1}^{n}[\frac{1}{m}\sum_{y=1}^{m}{\left[I\left(x,y\right)-{\left\langle I\left(x,y\right)\right\rangle }_{y}\right]}^{2}], \end{array}$$ (3)

for kymographs with time axes ranging from 20 to 200 time frames. From the results we can see that the noise from kymographs aligned via WPAlign is independent of the number of time frames chosen during filming. However, kymographs aligned by the Reisner approach seem to undergo an increase in noise as the number of frames increases, until plateauing at roughly 120 frames.

We present a possible explanation for this result: as the number of time frames increases, the underlying DNA molecule is allowed more time to undergo conformational changes and random diffusive processes, rendering the first and last frames increasingly dissimilar. Thus any template frame chosen by the Reisner method will be increasingly dissimilar from the frames farthest from it in the kymograph. This renders the local stretching factor optimization more prone to becoming stuck in local minima for these “distantly related” frames, introducing noise in the final alignment. WPAlign, on the other hand, avoids this problem as it does not rely on the choice of a single template frame.

### Information Score

Before alignment, bright and dark features can stray into adjacent kymograph columns as the DNA molecule undergoes horizontal diffusion. Thus thin features are obscured in the time average, effectively broadening peaks and valleys. As alignments improve, however, features occupy less horizontal space and become more apparent in the final time-trace. Since these features are essential in later performing statistical comparisons with other data, increasing contrast between neighboring features (i.e., increasing feature sharpness) essentially increases the information contained in the time trace.

To quantify this information content of the barcodes we present a new scheme based on the self-information of a random variable [42], see Methods for a comprehensive description. Here in brief, the information score, IS, of a kymograph is given by

$$\begin{array}{c} \text{IS}=\sum_{k}-\log[\frac{1}{\sqrt{2\pi \log(\sigma^{2}+\chi )}}\exp\{-\frac{\log{\left(\left|\Delta I_{k}\right|\right)}^{2}}{2\log(\sigma^{2}+\chi )}\}] \end{array}$$ (4)

where *σ* ^2^ represents the noise of the underlying kymograph, *χ* = 1 is a regularization parameter ensuring that the score remains real-valued for all noise levels, and the Δ*I*’s represent “robust” intensity differences (see S1) between neighboring peaks and valleys. Thus information increases as the difference between neighboring peaks and valleys grows and as the noise of the underlying kymograph decreases. That is, time-traces with sharper, more well-defined local extrema will contain more information than time traces from noisy kymographs with broad extrema.

**Fig 10:**
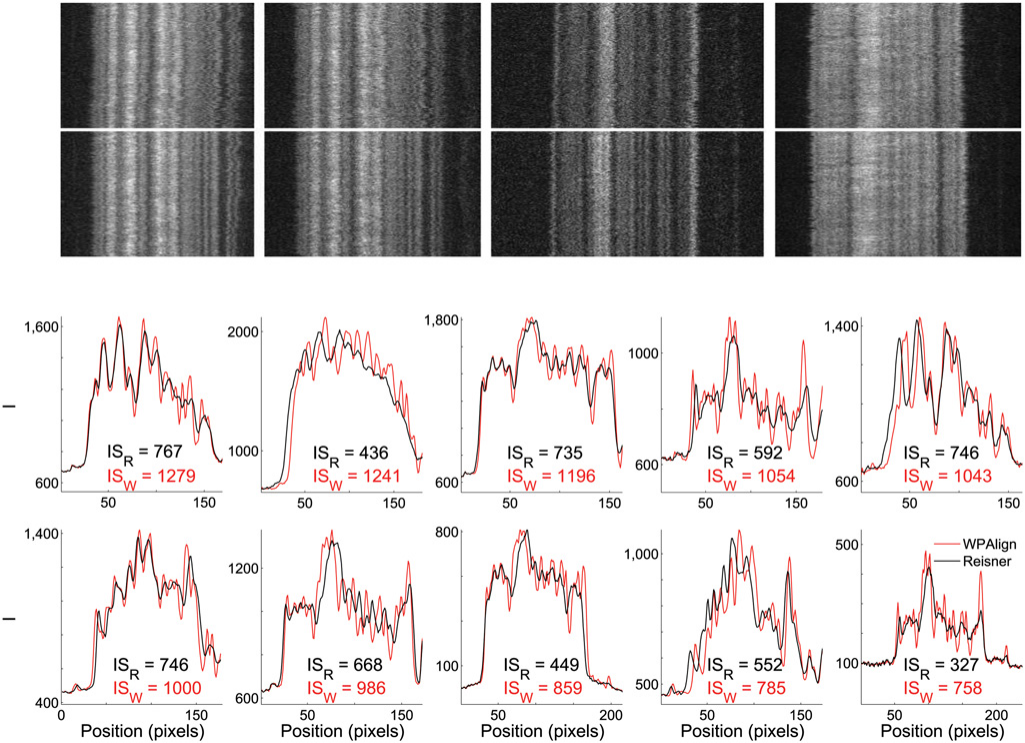
Quality comparison of barcodes aligned via the Reisner approach and WPAlign. (Top) Representative kymographs aligned via the Reisner approach (above) and WPAlign (below). (Bottom) Average intensity traces from kymographs in Fig 7 aligned via WPAlign (red) and the Reisner approach (black). The information score is displayed below each trace for both methods, quantifying the contrast between neighboring features. Notably, ⟨(IS_*W*_ − IS_*R*_)/IS_*R*_⟩ = 0.78, indicating that WPAlign on average produced time-traces with slightly more information than the Reisner method over our sample set. Note that the plots are in the same order as the corresponding kymographs in Fig 7 and are ordered by decreasing IS_*W*_.

Time traces and their corresponding information scores were calculated for the representative kymographs shown in Fig 7 after undergoing alignment by both WPAlign and the Reisner method (see Fig 10). In general, WPAlign produces visually sharper and more well-defined time-traces. Furthermore, the information content is greater for WPAlign in all examples considered. In fact, the average information gain from using WPAlign over the Reisner method on our dataset is roughly 78% (i.e., ⟨(IS_*W*_ − IS_*R*_)/IS_*R*_⟩ = 0.78).

Furthermore, independent of our kymograph alignment technique, this information score can serve as an objective and easily interpretable barcode quality measurement by which barcodes can be compared, providing a basis for experimental optimization. For example, expected barcodes can be calculated from theory for a number of experimental conditions [18], and then experiments can be performed only for barcodes expected to yield the highest information content, saving valuable time and resources for experimentalists. Furthermore, if theory is not available for a particular optical mapping application, the information score can serve as a quality criterion for experimental barcodes by which experimental conditions can be rigorously optimized.

### Computational Time

**Fig 11:**
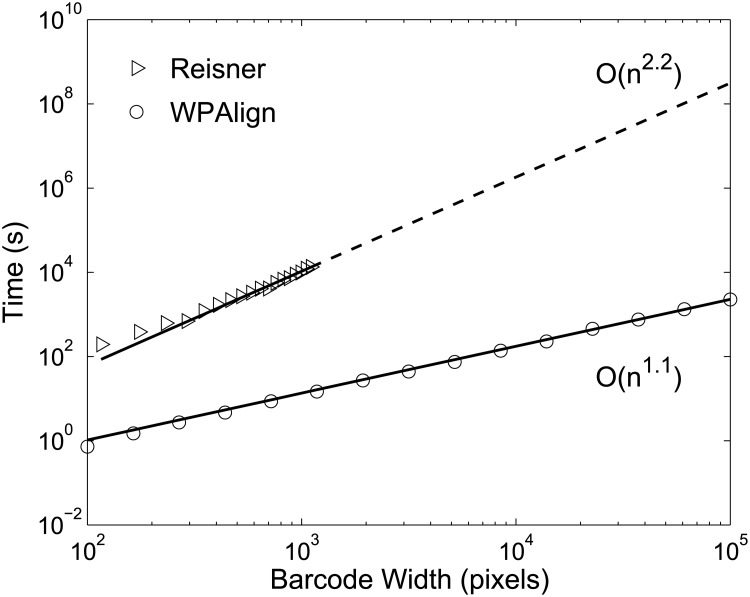
Comparison of time scaling for WPAlign and the Reisner approach. Empirical time scaling results for both techniques on identical kymographs ranging between ∼ 10^2^ and ∼ 10^5^ pixels in width, where ∼ 10^5^ pixels is roughly the length of an intact human genome at current resolutions. Power law curves of the form *ax* ^*b*^ were fit to these data (solid lines), yielding *b* = 1.1 for WPAlign, and *b* = 2.2 for the Reisner method. Thus WPAlign exhibits approximately *O*(*n*) time scaling with barcode width, while the Reisner method scales approximately with *O*(*n* ^2^). Scaling data beyond ∼ 10^3^ pixels was projected for the Reisner approach (dashed line), as alignment times became prohibitive. Simulated kymographs were generated by concatenating experimental T4GT7 kymographs from above.

Empirically, WPAlign exhibits linear scaling with barcode length, *n* (see Fig 11). This is somewhat intuitive, as the bottleneck of the approach is the shortest path finding algorithm, and this runs in linear time due to the directed and acyclic qualities of our networks. The Reisner approach, on the other hand, scales with *O*(*n* ^2^), rendering it impractical for bacterial barcodes on the order of 1 Mbp or larger (see Fig 11).

Perhaps most importantly, WPAlign was able to successfully align a simulated barcode with length on the order of a full human genome in only 40 minutes. The Reisner approach would require over 10^8^ seconds, or roughly 3 years, to perform this task on an identical computer.

## Discussion

In this paper we present a new DNA barcode kymograph alignment algorithm which outperforms an existing alternative [18] in computational speed, and for the particular data presented here, also slightly improves on noise reduction properties and information content of the time-averaged barcodes. Indeed, the orders-of-magnitude improvement in computational speed could open the door for high throughput kymograph alignment at the human-genome scale as well as constituting an important step in data analysis for a number of nanofluidic optical mapping techniques, including denaturation mapping [18], fluorocoding [19], competitive binding [20, 21], and enzymatic nicking [23, 24]. By providing a rapid framework for this data analysis, WPAlign can help bring the many applications of optical mapping closer to application, including bacterial strain and species identification, detection of large-scale genomic structural variation, and scaffold building for third generation *de novo* sequencing techniques. Furthermore, the algorithm is easy to implement, as the various substeps involved are available in most standard numerical packages.

Moreover, our feature detection method, suitably modified, may find application in other domains of biological image analysis, such as automated organism tracking [43, 44], or automated study of cellular transport along axons (à la [45–48]), defects in which have been implicated in a number of neurodegenerative diseases, including Alzheimer’s Disease, Parkinson’s Disease, and Amytrophic Lateral Sclerosis [49, 50].

Finally our information score, motivated as a way to compare the quality of alignments produced by different methods, may find widespread application among the optical mapping community as an easily calculable and interpretable barcode quality criterion by which to optimize experimental conditions.

## Supporting Information

S1 Methods Details on the algorithms presented herein. (PDF)
