# Supplementary material for: A Fast and Scalable Kymograph Alignment Algorithm for Nanochannel-Based Optical DNA Mappings
Source: PLoS One. 2015 Apr 13;10(4):e0121905. doi: 10.1371/journal.pone.0121905 (PMC4395267; doi:10.1371/journal.pone.0121905)
Supplement: S1 Methods — (PDF) [file pone.0121905.s001.pdf]

Supporting Information S1: A fast and scalable kymograph alignment algorithm for nanochannel-based optical DNA mappings

Charleston Noble[1,2,4], Adam N. Nilsson[1], Camilla Freitag[2,3], Jason P. Beech[2], Jonas O. Tegenfeldt[2], Tobias Ambjörnsson[1,*]

1 Department of Astronomy and Theoretical Physics, Lund University, Lund, Sweden

2 Division of Solid State Physics, Department of Physics, Lund University, Lund, Sweden

3 Department of Physics, Gothenburg University, Gothenburg, Sweden

4 Current address: Department of Systems Biology, Harvard Medical School, Boston, MA 02115, USA

∗ **E-mail: tobias.ambjornsson@thep.lu.se**

Kymograph Generation

As raw data, we are given movies of fluorescently stained DNA molecules confined in nanochannels. To generate kymographs from these movies, we perform the following procedure. First, we create a single representative frame via a simple time average of the entire movie. This frame is then rotated so that the channels are horizontal, and individual molecules are detected via image segmentation. For a given molecule, a 1D intensity trace is calculated for each movie frame by averaging over a 3-pixel vertical window, and these 1D traces are then stacked to form a kymograph.

The Reisner Method

Here we explain our implementation of the Reisner alignment method, as described in [1]. As usual, we begin with a raw, unaligned kymograph produced as detailed above. To begin the alignment, a template row, T, is chosen (typically picked near the middle of the kymograph), and then each non-template row, A_i, is individually and independently aligned to the template row in the following way.

First, all the A_i are translated linearly so that their centers of mass are aligned to T's. Then the next step is to "smooth out" the local longitudinal fluctuations. This is done by dividing the A_i into a series of uniform length pieces and applying a set of dilation/contraction factors to them.

In practice, a piecewise linear map S_i is created, and the slopes of the individual linear components are defined by dilation factors d_k. Thus S_i itself is a function of the d_k, for example $S_i(d_k)$. Now this

map S_i operates on the row A_i we wish to align, and a new profile, $A_i^{'}(x_j, d_k)$ is created (where x_j is the jth pixel in the profile). The parameters d_k are chosen to minimize the least squared difference Δ between the row A_i and the template row T:

$$\Delta = \sum_{j=1}^{N} \left[A_i^{'}(x_j, d_k) - T(x_j) \right]^2$$

Minimizing Δ with respect to the d_k can be performed by any standard global optimization procedure; however it must be noted that the process is very susceptible to local minima. Thus in our implementation we employ simulated annealing; in this process we limit the number of Δ evaluations to a number which increases linearly with the number of dilation factors d_k.

Once the dilation factors have been chosen for all rows, they are normalized so that the average dilation d_k across all rows is one (i.e., $\langle d_k(i) \rangle = 1$). This is done to better approximate the true equilibrium conformation of the DNA and help minimize the effect of template frame choice. Now the $d_k(i)$ are applied to all rows A_i to obtain the final aligned kymograph.

Laplacian of Gaussian Filter

The Laplacian of Gaussian filter is a standard image processing technique which is useful for "blob" detection. It uses the sum of second derivatives in the image to emphasize blobs of size roughly given by the variance of the Gaussian kernel. In this way, we emphasize not so much edges as ridges and valleys in the data which will be easier to detect in our feature detection step.

To perform the Laplacian of Gaussian, first we convolve I with a Gaussian kernel

$$g(x, y, t) = \frac{1}{2\pi t} \exp\left\{ -\frac{x^2 + y^2}{2t} \right\} \tag{1}$$

to give a scale space representation $L(x, y; t) = g(x, y, t) \star I(x, y)$. Then the Laplacian operator $\nabla^2 L = L_{xx} + L_{yy}$ is computed, resulting in strong positive responses for dark regions of extent $\sqrt{2t}$ and strong negative responses for bright regions of similar extent [2]. For our data, we have found $t = 10$ pixels to be adequate.

The size of the applied filter is set to be 10 pixels in the horizontal direction and only 3 pixels in the vertical direction, rendering the process close to one dimensional but with a small vertical component.

This is done because features are expected to fluctuate horizontally between vertical time frames.

Calculating K_D and K_B

Given a Laplacian response $K(x, y)$, linearly scaled such that each value falls in the range $[-1, 1]$, we calculate K_D and K_B using

$$K_D(x,y) = \begin{cases} 1 - K(x,y) & \text{for } K(x,y) > 0 \\ B & \text{for } K(x,y) \leq 0 \end{cases} \tag{2}$$

$$K_B(x,y) = \begin{cases} 1 + K(x,y) & \text{for } K(x,y) < 0 \\ B & \text{for } K(x,y) \geq 0 \end{cases}, \tag{3}$$

where $B \gg 1$ is a large constant which creates "barrier" pixels through which paths will not traverse. In this way, we prevent the feature detection algorithm from "jumping" between adjacent features. Then in K_D, small values represent dark regions, and in K_B small values represent bright regions.

Information Score

Here we introduce an information score associated with a DNA barcode, given as a 2D intensity profile, I. This score was chosen to quantify the amount, and sharpness, of "robust" peaks and valleys in the barcode.

We define these "robust" extrema as those which differ from neighboring pixels by an amount greater than a threshold value, I_{th}, typically chosen to be equal to the background noise level. In order to quantify this background noise, we assume that I has already been aligned. By the nature of the alignment, each column in I represents a single intensity value obscured by the addition of noise due to the photophysics of the dyes, noise in the imaging system and thermal fluctuations of the confined DNA molecules. Thus we create a new image whose pixel in the yth row, xth column, is given by

$$I'(x,y) = I(x,y) - \langle I(x,y) \rangle_y, \tag{4}$$

where $I(x, y)$ is simply the intensity of this pixel in the aligned kymograph, and $\langle I(x, y)\rangle_y$ is the mean

intensity of the xth column of the kymograph. Then, assuming perfect alignment, I' is an image with intensities attributable only to our kymograph's background noise, so our background variance σ^2 is given by the intensity variance of I', or

$$\sigma^2 = \langle (I' - \langle I' \rangle)^2 \rangle. \tag{5}$$

Now we calculate the time average of I, denoted $\langle I(x,y)\rangle_y$, given by

$$\langle I(x,y)\rangle_y = \frac{1}{m}\sum_{y=1}^{m} I(x,y) \tag{6}$$

and we locate the "robust" peaks and valleys by treating $\langle I(x,y)\rangle_y$ as an energy landscape and employing the method of Azbel, developed for a simplified description of the random melting of a one dimensional two-component Ising model [3, 4] (see below). Thus we obtain only local extrema which are "robust," i.e., regions which to the left and right are surround by an "energy barrier" larger than the threshold $I_{\rm th}$ (typically chosen equal to the average background noise, i.e., σ).

Let us now, for completeness, briefly review the method by Azbel [3, 4] for finding robust local maxima and minima. The method scales linearly with the barcode length and uses the fact that robust local maxima and minima are alternating, i.e. a local maximum must be followed by a local minimum and vice versa. Since the barcode's initial pixels in general represent background noise, in our version of the Azbel approach we utilize that the first local extremum must be a minimum. The algorithm now proceeds as follows: we step through the pixels, x, consecutively. For each pixel we calculate the intensity difference $C(x_s, x) = \langle I\rangle_x - \langle I\rangle_{x_s}$ where x_s is a start pixel as defined below. At the start of the algorithm we set $x_s = 1$ and calculate $C(x_s, x)$ for increasing x as long as the criteria $C(x_s, x) < I_{\rm th}$ is fulfilled (implemented through a while-loop). Once this criterion is violated we know that the pixel x_s must be a robust local minimum (surrounded to the right by a barrier larger than $I_{\rm th}$), so this pixel number is stored and the while-loop terminated. If, within the while-loop above, the intensity difference decreases, i.e. we find that $C(x_s, x) < 0$, we must have a new local minima and we shift x_s to the present pixel position, $x_s \to x$, with a corresponding reset of $C(x_s, x)$. Once a robust local minima has been identified according the the scheme above, we proceed in an identical fashion to identify a subsequent robust local maximum, starting at pixel x. To this purpose we invoke the condition $C(x_s, x) > -I_{\rm th}$ (and $C(x_s, x) > 0$ as a requirement for shifting x_s) within a new while-loop. The procedure above is repeated until all n pixels representing $\langle I(x,y)\rangle_x$ has been exhausted. In Fig. S1 we display the local extrema found using

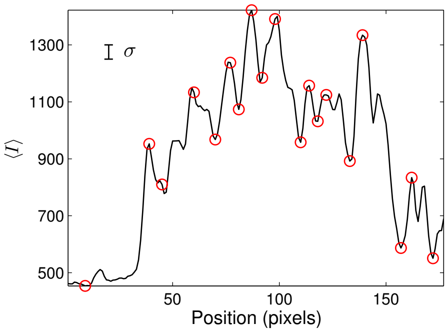

Figure S1. Illustration of "robust" extrema detected by Azbel's method. The aligned time-average of kymograph 6 (from Fig. 7 in the main text) was used, with $\sigma = 50$ intensity units for illustrative purposes.

the approach above.

Finally our information score is that of *self information* [5], and we define the information content IS of a DNA barcode by

$$\text{IS} = \sum_k -\log\left[\frac{1}{\sqrt{2\pi\log(\sigma^2+\chi)}}\exp\left\{-\frac{\log(|\Delta I_k|)^2}{2\log(\sigma^2+\chi)}\right\}\right], \tag{7}$$

where the ΔI_ks are the intensity differences between neighboring peaks and valleys identified above, and k denotes the numbering of ΔIs found. Also, $\chi = 1$ is a regularization parameter which ensures that IS is real-valued for all noise levels.

To justify the use of the logarithm of $\Delta I(k)$ rather than $I(k)$ itself in Eq. (7) we note that in a simplistic approach to DNA melting we have that the probability for a DNA basepair to be open is related to the Boltzmann weight $P(i) \propto \exp(-\beta\Delta E(i))$, where $\Delta E(i)$ is the energy difference between a basepair being open and closed. Therefore, by using $\log[I(k)]$ our information score utilizes free energy differences.

Experimental Procedure

To generate the optical DNA mappings shown in the main text, we employed the following experimental protocol. T4GT7 DNA (supplied by Nippon Gene, Japan through Wako Chemicals GmbH, Neuss, Germany) was mixed at a ratio of 1 dye molecule per 6 base pairs with YOYO1® Iodide (LifeTechnologies®,

USA) and kept at 50°C for 2 hours to ensure homogeneity of staining. Melting experiments were performed in a buffer consisting of 10mM NaCl in 0.05 x TBE (1 x TBE is 89mM Tris, 89mM Boric acid and 2mM EDTA). Beta-mercaptoethanol was added to a final concentration of 2% and formamide to a final concentration of 50%.

Using compressed nitrogen the buffer carrying the DNA molecules was forced into nanochannels with cross section 100 nm by 150 nm etched in fused silica. Once in the nanochannels the molecules were imaged in a Nikon TE2000 microscope (Nikon, Tokyo, Japan) using a high-pressure mercury lamp for excitation, a FITC filter cube to pick out the fluorescence from the stained DNA and an Andor Ixon DU 897 EMCCD (Andor Technology, Belfast, Ireland) camera for image acquisition.

In order to form the melt maps the nanochannel device was brought into contact with an aluminium block, heated to 31.5°C at which temperature the molecules are partially denatured. We utilized the time dependence of the barcode formation process to create kymographs of different quality and different information contents. Molecules were imaged over several distinct 5-minute periods resulting in the 10 kymographs in Fig. 7 in the main text, representing a collection of 4 molecules. Kymographs generated from the same molecules were: (1, 2, 5), (3, 8), (7, 4, 10), (6, 9).

References

1. Reisner W, Larsen NB, Silahtaroglu A, Kristensen A, Tommerup N, Tegenfeldt JO, et al. Single-molecule denaturation mapping of DNA in nanofluidic channels. Proceedings of the National Academy of Sciences 2010;107: 13294–13299.

2. Lowe DG. Distinctive image features from scale-invariant keypoints. International journal of computer vision 2004;60: 91–110.

3. Azbel MY. Random two-component one-dimensional ising model for heteropolymer melting. Physical Review Letters 1973;31: 589–592.

4. Azbel MY. DNA sequencing and helix–coil transition. i. theory of dna melting. Biopolymers 1980;19: 61–80.

5. Cover TM, Thomas JA. Entropy, relative entropy and mutual information. Elements of Information Theory 1991: 12–49.
